# Predictive Relevance of Baseline Lactate and Glucose Levels in Patients with Spontaneous Deep-Seated Intracerebral Hemorrhage

**DOI:** 10.3390/brainsci11050633

**Published:** 2021-05-14

**Authors:** Felix Lehmann, Lorena M. Schenk, Matthias Schneider, Joshua D. Bernstock, Christian Bode, Valeri Borger, Florian Gessler, Erdem Güresir, Alexis Hadjiathanasiou, Motaz Hamed, Marcus Müller, Christian Putensen, Julian Zimmermann, Hartmut Vatter, Patrick Schuss

**Affiliations:** 1 Department of Anesthesiology and Intensive Care, University Hospital Bonn, 53127 Bonn, Germany; christian.bode@ukbonn.de (C.B.); christian.putensen@ukbonn.de (C.P.); 2 Department of Neurosurgery, University Hospital Bonn, 53127 Bonn, Germany; lorena_maria.schenk@ukbonn.de (L.M.S.); matthias.schneider@ukbonn.de (M.S.); valeri.borger@ukbonn.de (V.B.); erdem.gueresir@ukbonn.de (E.G.); alexis.hadjiathanasiou@ukbonn.de (A.H.); motaz.hamed@ukbonn.de (M.H.); hartmut.vatter@ukbonn.de (H.V.); patrick.schuss@ukbonn.de (P.S.); 3 Department of Neurosurgery, Brigham and Women’s Hospital, Harvard Medical School, Boston, MA 02115, USA; jbernstock@bwh.harvard.edu; 4 Department of Neurosurgery, University Hospital Rostock, 18057 Rostock, Germany; florian.gessler@med.uni-rostock.de; 5 Department of Neurology, University Hospital Bonn, 53127 Bonn, Germany; marcus_mathias.mueller@ukbonn.de (M.M.); julian.zimmermann@ukbonn.de (J.Z.)

**Keywords:** intracerebral hemorrhage, lactate, glucose, mortality

## Abstract

(1) Background: As elements of the standard admission blood panel, lactate and glucose represent potential biomarkers for outcome prediction. In patients with intracranial hemorrhage (ICH), data on the predictive value of these blood values is exceedingly sparse. (2) Methods: Between 2014 and August 2020, all patients with deep-seated ICH referred to the neurovascular center at the authors’ institution were included in the subsequent study. Serum levels of lactate and glucose at the time of admission were compared with mortality at 90 days. In addition, a multivariate analysis was performed in order to identify independent admission predictors for 90-day mortality. (3) Results: Among the 102 patients with deep-seated ICH, elevated lactate and glucose levels on admission were significantly associated with increased mortality at 90 days. Multivariate logistic regression analysis identified “ICH score ≥3” (*p* = 0.004) along with “admission hyperlactatemia” (*p* = 0.025) and “admission hyperglycemia” (*p* = 0.029) as independent and significant predictors of 90-day mortality in patients with deep-seated ICH. (4) Conclusions: Initially elevated lactate and glucose levels after spontaneous intracerebral hemorrhage are associated with poor outcome, suggesting a potential application for future prognostic models when considered in conjunction with other parameters.

## 1. Introduction

Spontaneous intracerebral hemorrhage (ICH) is a potentially devastating neurological emergency in which long-term functional independence is achieved in only a fraction of cases and mortality rates are tremendous, exceeding 50% [1]. Therefore, prognostic factors for mortality after ICH are warranted for early assessment of the potential success/necessity of an often-debilitating intensive care/surgical treatment [2]. Of particular value in this context are clinical predictors that are easily available at admission after spontaneous ICH and are also not subject to increased interobserver variability (such as measures of ICH size). Promptly available biomarkers on admission may support early risk assessment of a complicated course and provide additional insight into pathophysiological mechanisms if such factors are causally related to the event or its immediate sequelae [3]. In critically ill patients, initially elevated serum lactate levels have previously been identified as associated with an unfavorable outcome [4,5]. In contrast, the prognostic value of blood lactate levels in ICH patients has been studied scarcely. Whereas several studies have indicated that glucose is associated with outcome in ICH [6,7,8,9]. Lactate and glucose represent two crucial metabolites that are also interrelated. Glucose constitutes a direct precursor of lactate, and both circulating blood levels of lactate and glucose can be increased by different stress conditions [10]. An issue with studies of spontaneous ICH is the distinct heterogeneity due to the varying location, size, and sequelae of hemorrhage [11]. Therefore, the present study focused exclusively on patients with spontaneous non-traumatic ICH in the area of the basal ganglia (herein: deep-seated ICH). The aim of this study was to determine whether early elevations in circulating lactate and glucose levels are associated with increased mortality after spontaneous deep-seated ICH.

## 2. Materials and Methods

Patients: For the present retrospective analysis, all patients with spontaneous deep-seated ICH admitted to the neuro-intensive care unit (NICU) of the author’s institution between 2014 and August 2020 were considered to be eligible for potential inclusion in the present study. Patients with lobar ICH and/or an underlying source of bleeding (e.g., aneurysm, AVM, trauma) were excluded. Patients who were not treated in the NICU for at least 3 days and those whose disastrous clinical situation (e.g., intractable cardio-/pulmonary instability, brain injury not deemed compatible with survival) precluded further meaningful intensive care were also excluded. Patients with an existing patient wish for withdrawal of life-sustaining treatment were not considered in further study analysis. Baseline demographic and clinical data, including age, sex, vital signs, radiological features, length of stay, and mortality, were retrospectively obtained from medical records. The results of the routine blood tests performed on admission and in the subsequent 4 days of treatment were reviewed with regard to serum lactate level and blood glucose level. Hyperlactatemia at the time of hospital admission was defined as a lactate value of >1.8 mmol/L and hyperglycemia as a glucose value of >180 mg/dL according to institutional and described reference values [12]. In addition, the highest values of the first 5 days of treatment were determined and analyzed separately as peak lactate and/or peak glucose levels. Unaware of patient outcome, initial head computed tomography (CT) scans were used to measure both the presence of intraventricular blood and hematoma size (ABC/2 method, [13]). The initial degree of affected consciousness was determined by means of the Glasgow Coma Scale (GCS). With the data collected the ICH score was calculated for each individual patient [14]. For further statistical analysis, patients were divided into two groups based on ICH score: (1) ICH score < 3 and (2) ICH score ≥ 3. All patients were treated according to the standard guidelines [15], and the primary end point of the present study was all-cause mortality at 90 days. Follow-up was obtained 3 months after ictus. Therefore, functional outcome was assessed at 3 months after ICH using the modified Rankin Scale (mRS). The affected patients were consequently divided into two groups, where, also taking into account the eloquence of the ICH localization, favorable outcome was defined as mRS 0–4, while unfavorable outcome was defined as mRS 5–6. For clarity of presentation, patients were assigned to the non-survivor group or the survivor group according to mortality within 90 days after hemorrhage.

Statistics: All results are presented as medians with interquartile range (IQR) for continuous variables and as numbers with percentages for categorical variables. Fisher’s exact test was applied to compare unpaired categorical and binary variables. The Mann–Whitney U-test was chosen to compare continuous variables as the data were largely not normally distributed. Receiver operating characteristic (ROC) curve analysis was also conducted, and the area under the curve (AUC) values were calculated to evaluate the utilization of serum lactate levels as well as glucose levels for mortality prediction in patients with deep-seated ICH. All statistical analyses were performed using the computer software package SPSS (version 25, IBM Corp., Armonk, NY). Results with *p* < 0.05 were considered statistically significant. Furthermore, a multivariate analysis was performed to confirm the independence of the potential outcome predictors studied. The following variables were included in a multivariate logistic regression model applying the events per variable (EPV) 1 to 10 rule of thumb: ICH score ≥ 3, admission hyperlactatemia, admission hyperglycemia, and anticoagulant medication prior ictus.

## 3. Results

### 3.1. Patient Characteristics

A total of 1409 patients with the diagnosis of ICH between 2014 and August 2020 were referred to the authors’ institution. After implementing the aforementioned inclusion criteria (spontaneous, non-traumatic, supratentorial, deep-seated ICH with NICU stay ≥3 days), 102 patients with deep-seated ICH were identified as eligible for further analysis. Median age of patients 66 years (IQR 57–76). Mortality rate after 90 days was 42% (43/102). Overall, 57 patients (56%) achieved a favorable outcome (mRS 0–4), whereas 45 patients with deep-seated ICH (44%) experienced an unfavorable outcome (mRS 5–6). Baseline characteristics of patients with deep-seated ICH are given in Table 1.

### 3.2. Influence of Early Serum Lactate on Mortality

**Figure 1:**
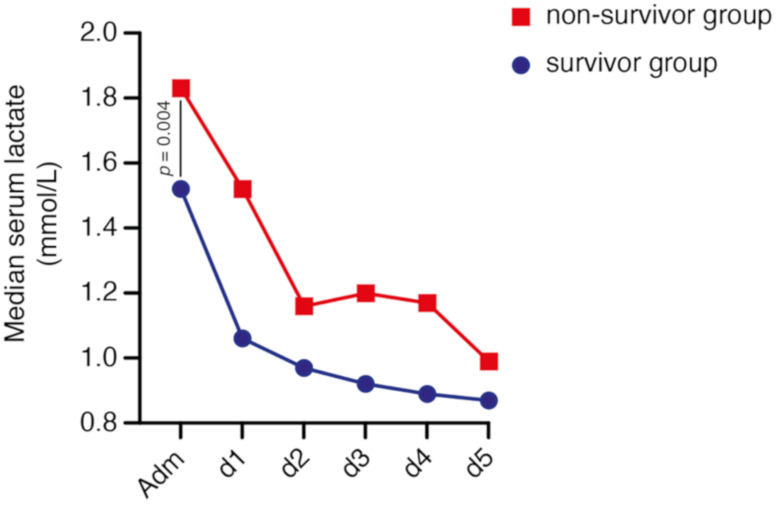
Chronological progression of lactate values distributed according to mortality at 90 days in patients with deep-seated intracerebral hemorrhage within the initial period after admission.

Overall, median admission serum lactate level was 1.56 (IQR 1.18–2.16) mmol/L in all patients. Forty-one patients (40%) presented with hyperlactatemia on admission. In detail, 16 patients with deep-seated ICH who survived (27%), compared to 25 patients of the non-survival group (58%), presented with initial hyperlactatemia (*p* = 0.002, OR 3.7, 95% CI 1.6–8.6). Median admission serum lactate level was 1.48 (IQR 1.05–1.88) mmol/L in the group of patients with deep-seated ICH who survived and 1.88 (IQR 1.49–2.41) mmol/L in the non-survival group (*p* = 0.004). Patients with deep-seated ICH categorized in the survivor group exhibited a significantly lower median peak lactate value (1.51 mmol/L, IQR 1.19–2.14), compared to patients allocated to the non-survivor group (2.08 mmol/L, IQR 1.59–2.70; *p* = 0.001). The course of the median serum lactate values of the initial five days after admission are shown in Figure 1.

**Figure 2:**
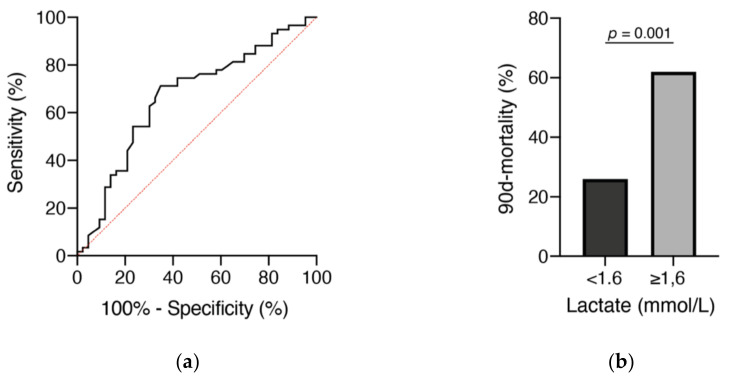
Receiver operating characteristic (ROC) curves (**a**) for admission lactate levels and mortality after 90 days in relation to initial lactate levels (**b**).

Regarding the initial serum lactate value, the optimal cutoff was 1.6 mmol/L and the AUC was 0.67 (95% CI 0.56–0.78, *p* = 0.004), with a sensitivity of 67% and a specificity of 71% (Figure 2a). In patients who presented with serum lactate < 1.6 mmol/L, mortality rate after 90 days was 26% (15/57) compared to 62% in patients with an initial serum lactate ≥ 1.6 mmol/L (28/45; *p* = 0.001, OR 4.6, 95% CI 1.99–10.7, Figure 2b).

### 3.3. Influence of Admission Glucose on Mortality

**Figure 3:**
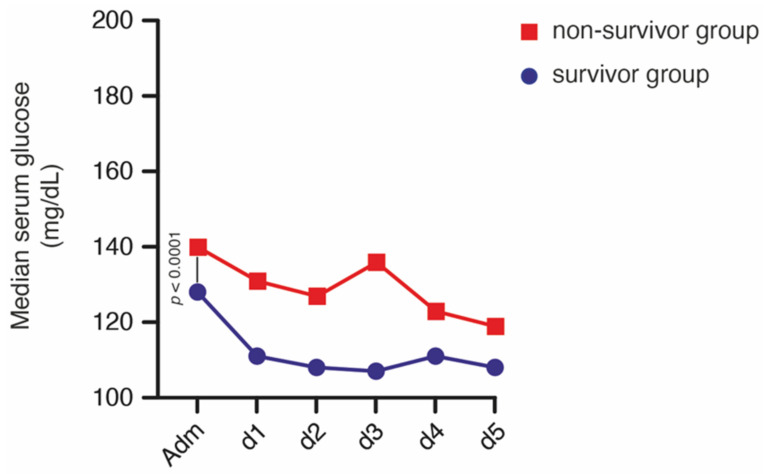
Chronological progression of blood glucose values distributed according to mortality at 90 days in patients with deep-seated intracerebral hemorrhage within the initial period after admission.

Overall, median admission glucose level was 130 (112–148) mg/dL in all patients. Hyperglycemia was detected on admission in 15 patients (15%). In detail, three patients with deep-seated ICH who survived (5%) compared to 12 patients of the non-survival group (28%) presented with initial hyperglycemia (*p* = 0.002, OR 7.2, 95% CI 1.9–27.6). Median admission glucose level was 126 (IQR 112–132) mg/dL in the group of patients with deep-seated ICH who survived and 143 (IQR 120–183) mg/dL in the non-survival group (*p* = 0.001). Patients with deep-seated ICH categorized in the survivor group exhibited a significantly lower median peak lactate value during the study time period (130 mg/dL, IQR 121–144) compared to patients allocated to the non-survivor group (163 mg/dL, IQR 133–213; *p* < 0.0001). The course of the median glucose values of the initial five days after admission are shown in Figure 3.

**Figure 4:**
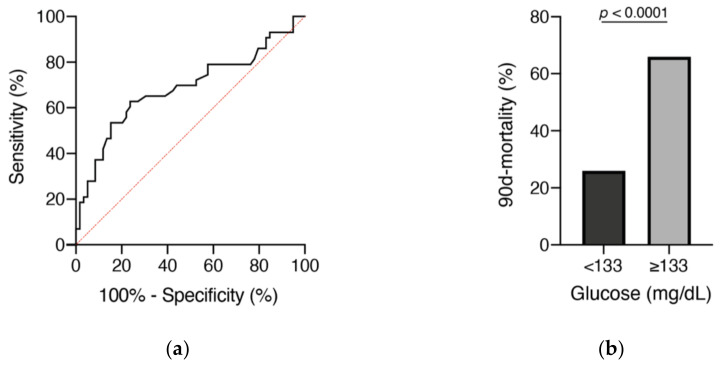
Receiver operating characteristic (ROC) curves (**a**) for admission blood glucose levels and mortality after 90 days in relation to initial blood glucose levels (**b**).

Regarding the initial glucose value, the optimal cutoff was 133 mg/dL and the AUC was 0.69 (95% CI 0.58–0.796, *p* = 0.001), with a sensitivity of 63% and a specificity of 76% (Figure 4a). In patients who presented with admission glucose < 133 mg/dL, mortality rate after 90 days was 26% (16/61), compared to 66% in patients with an initial glucose ≥ 133 mg/dL (27/41; *p* < 0.0001, OR 5.4, 95% CI 2.3–12.8; Figure 4b).

### 3.4. Multivariate Analysis

The additionally performed multivariate logistic regression analysis identified “ICH score ≥ 3” (*p* = 0.004, OR 6.4, 95% CI 1.8–22.7), “admission hyperlactatemia” (*p* = 0.025, OR 2.9, 95% CI 1.1–7.4), and “admission hyperglycemia” (*p* = 0.029, OR 5.0, 95% CI 1.2–21.3) as independent and significant predictors for 90-day mortality in patients with deep-seated ICH (Nagelkerke’s R^2^ 0.329).

## 4. Discussion

Critically ill patients after spontaneous intracerebral hemorrhage often have to undergo extensive therapies. Early reliable biomarkers to assess mortality might help in the course of this often-sophisticated management of affected patients to support the assessment of benefit and/or necessity of specific intensive medical therapy (organ replacement procedures) or neurosurgical interventions (decompressive hemicraniectomy) [2,3,16]. In patients with deep-seated ICH, the present study demonstrates an association between an initial elevated lactate concentration, as well as an elevated glucose level, with an increased likelihood of mortality at 90 days after the ictus. 

In patients with spontaneous ICH, an initially elevated serum lactate level might reflect a multifactorial pathophysiology. These patients often experience a catecholamine response due to hemorrhage-related hypothalamic dysfunction with the possible consequence of tissue hypoxia from pulmonary edema [17,18]. Due to this sudden catecholamine release combined with a sudden increase in intracranial pressure, enhanced renal perfusion may lead to subsequential hypovolemia [17]. In addition, excessive adrenergic stimulation could also induce increased glucose metabolism with rapid output of pyruvate and lactate, the latter being measurable in the systemic circulation. Similar observations have been made in patients with sustained aneurysmal subarachnoid hemorrhage [19,20]. In patients with deep-seated ICH, the results of the present case series indicate that both the existence of hyperlactatemia on admission as well as an exceeded peak level within the first days after hemorrhage seems to be linked to the probability of a decreased survival at 90 days. This spurs the importance of lactate-oriented therapy in critically ill patients, now including patients with deep-seated ICH in the present study [5].

Furthermore, experimental studies suggest that hyperglycemia after ICH may also be triggered by a neuroendocrine stress-mediated response and in turn aggravates subsequent brain tissue damage through metabolic dysregulation, cytotoxicity, and neuronal cell death [21,22]. In an animal experimental approach with induced hyperglycemia, Song et al. found it to result in more severe brain edema and perihematomal cell death [22]. Similarly, studies in the clinical setting-consistent with the present study-demonstrated that patients with elevated blood glucose on admission had significantly higher mortality at 90 days, regardless of preexisting glycemic status prior to hemorrhage [23]. The data of the present study also indicate, within patients with deep-seated ICH, that not only the presence of hyperglycemia on admission but also an excessive peak value occurring within the first days after hemorrhage seems to be associated with a lower probability of survival at 90 days. The guidelines for management of patients with ICH therefore state that hyperglycemia should be avoided due to its impact on outcome, not only during the course of treatment but also during the prehospital/admission period [15]. However, exaggerated correction of blood glucose into hypoglycemia should also be avoided to prevent energy crisis in such a vulnerable stage [15,24,25]. In the present study, only 12 patients (12%) had a history of diabetes mellitus. Similar to the study on initial glucose/lactate concentration in patients with aneurysmal subarachnoid hemorrhage by Ndieugnou Djangang et al., diabetes mellitus as a pre-existing condition did not appear as a decisive factor in the initial determination of mortality in patients with deep-seated ICH [19]. 

Thus, in a reasonably homogeneous patient population (deep-seated ICH), the present study indicates that an adequate, balanced therapy should be initiated early in response to the initial admission state. Moreover, these easily obtained laboratory parameters provide an early impression of the patient, which together with the radiological and neurological findings allows for an improved evaluation. 

This study has several limitations in addition to its retrospective design. The confinement to patients with deep-seated hemorrhage and assignment to a university medical center certainly offer advantages of reduced heterogeneity but do not allow an uncritical generalization of the results of this study to all patients with ICH. Furthermore, lactate and glucose were not measured at specific, predefined time points but were measured after admission. This circumstance neglects the different disease courses ahead of admission. Finally, we relied on systemic lactate and glucose values and had to forgo corresponding values from CSF and/or microdialysis.

## 5. Conclusions

Hyperlactatemia as well as hyperglycemia in the early phase of deep-seated intracerebral hemorrhage are associated with increased mortality. These findings add to the radiological/neurological risk profile of patients with deep-seated ICH, allowing earlier and more comprehensive assessment of individual management. 

## Figures and Tables

**Table 1:** Baseline patient characteristics.

	Survivors	Non-Survivors	*p*-Value
number of patients	59	43	
median age (IQR, yrs)	63 (53–72)	68 (59–81)	*p* = 0.03
female sex	21 (36%)	16 (37%)	*p* = 1.0
ICH score ≥ 3 at admission	4 (7%)	16 (37%)	*p* < 0.0001
initial GCS ≤ 12	28 (48%)	36 (84%)	*p* < 0.0001
ICH volume ≥ 30 mL	24 (41%)	29 (67%)	*p* = 0.009
known diabetes mellitus prior ictus	5 (9%)	7 (16%)	*p* = 0.4
anticoagulant medication prior ictus antiplatelet medication combination	8 (14%) 15 (25%) 1 (2%)	6 (14%) 13 (30%) 0 (0%)	*p* = 1.0
initial SBP (mmHg) mild (<180) moderate (180–219) severe (≥220)			
	36 (61%)	27 (63%)	
	17 (29%)	10 (23%)	*p* = 0.7
	6 (10%)	6 (14%)	
median admission lactate (IQR, mmol/L)	1.48 (1.05–1.88)	1.88 (1.49–2.41)	*p* = 0.004
median admission glucose (IQR, mg/dL)	126 (112–132)	143 (120–183)	*p* = 0.001

IQR, interquartile range; yrs, years; ICH, intracerebral hemorrhage; GCS, Glasgow Coma Scale; SBP, systolic blood pressure.

## Data Availability

All data are contained within the article.
